# Instability of Contact Resistance in MEMS and NEMS DC Switches under Low Force: the Role of Alien Films on the Contact Surface

**DOI:** 10.3390/s131216360

**Published:** 2013-11-28

**Authors:** Haodong Qiu, Hong Wang, Feixiang Ke

**Affiliations:** 1 NOVITAS, Nanoelectronics Centre of Excellence, School of Electrical and Electronic Engineering, Nanyang Technological University, Singapore 639798, Singapore; E-Mail: hdqiu@ntu.edu.sg; 2 Temasek Laboratories @ Nanyang Technological University (NTU), 50 Nanyang Drive, Research Techno Plaza, Singapore 637553, Singapore; E-Mail: kfeixian@hotmail.com

**Keywords:** MEMS, NEMS, contact instability, alien film, XPS, trap-assisted tunneling

## Abstract

The metal contact is one of the most crucial parts in ohmic-contact microelectromechanical (MEMS) switches, as it determines the device performance and reliability. It has been observed that there is contact instability when the contact force is below a threshold value (minimum contact force). However, there has been very limited knowledge so far about the unstable electrical contact behavior under low contact force. In this work, the instability of Au-Au micro/nano-contact behavior during the initial stage of contact formation is comprehensively investigated for the first time. It has been found that the alien film on the contact surface plays a critical role in determining the contact behavior at the initial contact stage under low contact force. A strong correlation between contact resistance fluctuation at the initial contact stage and the presence of a hydrocarbon alien film on the contact surface is revealed. The enhancement of contact instability due to the alien film can be explained within a framework of trap-assisted tunneling.

## 1. Introduction

Radio frequency microelectromechanical (RF MEMS) switches have been drawing a lot of research interest in the past two decades, due to their several advantages such as high isolation, low insertion loss, zero power consumption and high linearity [1]. RF MEMS devices can offer attractive alternatives in switching networks, portable wireless systems, phased arrays and so on [2]. On the other hand, the reliability of RF MEMS switches is a major concern for long term applications. There have been a number of reports in this area, including both experimental measurements [3–6] and computational simulations [7,8]. Generally, for capacitive switches, the lifetime is affected by the charging effects in the dielectric layer, and for ohmic-contact switches, the reliability is limited by the metal contacts.

**Figure 1.**
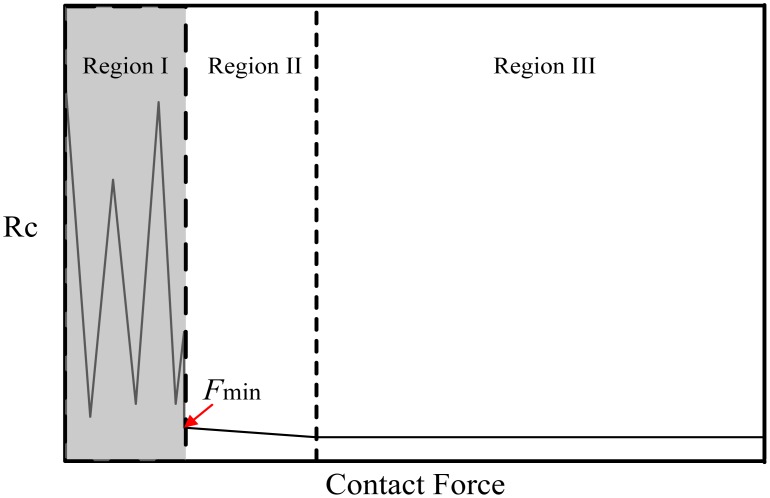
Schematic plot of contact resistance *R*_c_ *versus* contact force during contact making. Region I: unstable region; Region II: gradual reduction of *R*_c_; Region III: negligible reduction of *R*_c_.

Compared to bulk metals, the microscopic contact behavior remains an important still not fully understood topic. In micro-contacts, surface morphology has to be taken into consideration. The reliability and RF performance are closely related to the physical contact made between the prominent asperities at the contact surfaces. Load cycling tests have been performed for RF MEMS switches [3,9–12], to investigate the degradation mechanism of the metal contacts under different testing conditions. Meanwhile, the behavior of microscopic contacts during a single load test has been intensively studied, with the electrical contact resistance *R*_c_ as an important parameter to understand the contact behavior [13–16]. It was found that the relation between *R*_c_ and contact force can be divided into three regions, as shown in Figure 1.

A typical contact cycle starts from an unstable contact region (Region I) with a drastic fluctuation of the electrical contact resistance *R*_c_, followed by a stable but gradual reduction of *R*_c_ in Region II. Finally, it reaches a steady state with small *R*_c_ in Region III. A minimum contact force *F*_min_ is required to establish the stable electrical contact. *F*_min_ of 10 to 50 μN were reported in the literature for soft Au-Au contacts [13–15]. When the contact force exceeds *F*_min_ (Region II), the gradual reduction of *R*_c_ could be attributed to plastic deformation of surface asperities until the high force region (Region III), in which *R*_c_ is determined by film thickness effects on a macroscopic scale [14]. However, to the best of our knowledge, the unstable electrical contact behavior under low contact force (Region I) remains unexamined.

In conventional RF MEMS switches, the contact force ranges from tens to hundreds μN for Au-Au contacts [13,17], which is larger than *F*_min_ for most cases. For MEMS switches not using Au-Au contacts, the contact force is significantly higher (e.g., OMRON's switch with 5 mN per contact [18]). For RF applications, it is important to have a stable and low contact resistance. Therefore, past studies of contact behavior mainly focused on the stable region, including the load cycling tests. There is an increasing demand to scale down the MEMS components towards sub-micrometer dimensions for various applications such as NEMS logic gates and memories [19,20]. As a result, the contact force is drastically reduced to a value even smaller than 1 μN [2,19], which is far below the *F*_min_ reported in references [13] and [14] for Au-Au contacts. As a result, it is necessary to look into the unstable electrical contact behavior in the low contact force region.

On the other hand, for a stable metal-to-metal contact, it has been reported that the contact behavior is affected by the existence of an insulating alien film on the surface [9,11,12]. However, its role during the early stage of contact making has not been investigated due to the absence of characterization work [15]. Hermetic packaging is widely used to minimize the influence of the environment and improve reliability. The purpose of this work is to examine the unstable contact behavior of Au-Au micro/nano-contacts under low contact force, since Au has been considered as an important candidate for contact material in MEMS DC switches due to its low electric resistivity and resistance to surface oxidization. X-ray photoelectron spectroscopy (XPS) techniques are used for the analysis of the sample surfaces. The mechanism behind the instability of electrical contact resistance is discussed under a framework of trap-assisted tunneling. It should be pointed out that the main objective of this work is to identify the critical role of the alien film on the contact surface in determining the contact behavior under low contact force. The Au-Au contact is used as a test vehicle for the study. Pure Au contacts are not popular for low-force switches due to their poor reliability.

## 2. Experimental

**Figure 2.**
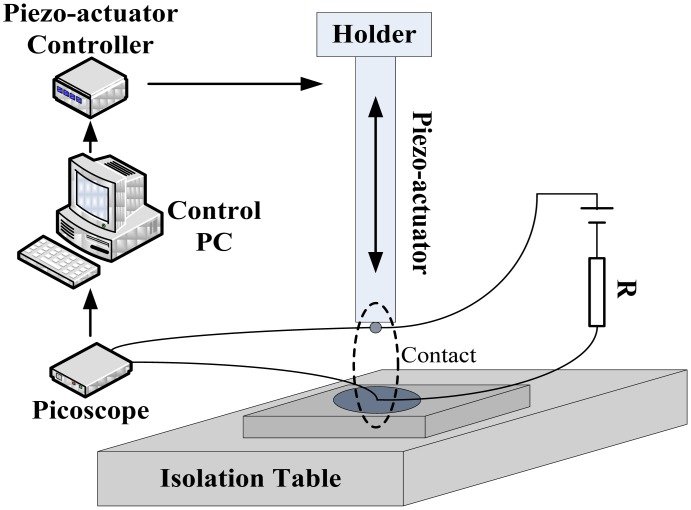
Schematic layout of the experimental apparatus.

A devised nanoindentation platform was applied to perform the experiments in this work (see Figure 2). The setup is built on an active optical table (T48W, Nexus, Newton, NJ, USA), to isolate the testing system from vertical and horizontal vibrations. A piezo-actuator (P-841.60, Physik Instrumente, Karlsruhe, Germany) is connected with PC workstation, which is able to produce smooth and continuous vertical motion within a range of a few nanometers repeatedly. The contact part uses a “ball-on-flat” configuration. The tip of the piezo-actuator is brought into contact with the sample placed on the *X*-*Y* stage during contact making; meanwhile, the changes in contact voltage *versus* loading time are captured by a digital storage oscilloscope (PicoScope 2204, Pico Technology, Cambridgeshire, UK), with a maximum sampling frequency of 100 MHz. Coaxial cables with bayonet neill–concelman (BNC) connectors are used for the connections, to minimize the delay time and avoid any possible electrical interference. Similar systems could be found in the literature for contact tests [14,15,21]. Fine control of the ball position with the piezo-actuator allows the tests to be performed under low contact force with high accuracy and repeatability.

High vacuum electron beam evaporation was used to coat gold film onto the ball tip of piezo-actuator and polished Si sample (2 inch) surface. A titanium film of 0.1 μm was deposited as an adhesive layer, followed by deposition of 1 μm gold film. The surface roughness of the coated gold film was determined by using atomic force microscopy (AFM). The root mean square (RMS) roughness obtained from 2 μm × 2 μm area is 4 nm. The ball tip and sample were cleaned by the standard cleaning procedures in clean room (5 min ultrasonic cleaning in acetone, isopropanol and deionized water sequentially, dried by nitrogen blower) before contact testing.

Two groups of samples (Group A and Group B) were used for contact study. Samples in Group A were treated as “fresh” samples and tested immediately after preparation, while samples in Group B were exposed in the MEMS fabrication environment for one complete lithography cycle using AZ photoresist (AZ 1518) before the contact tests, to mimic the surface condition of gold contact after microfabrication. AZ 1518 of 1.5 μm was spun on the sample followed by prebake at 100 °C for 60 s on a hotplate. After that, the samples underwent standard ultraviolet (UV) exposure and hotplate postbake (115 °C for 60 s). The photoresist was finally removed by acetone before the samples were loaded into the system for contact testing.

A large number of contact tests were performed under precisely controlled operational conditions. The tip displacement velocity of the piezo-actuator was fixed at (10 ± 0.9) nm/s, and the applied contact voltage varied from 80 to 300 mV. On the other hand, we took one sample from each group right before the contact tests, to study the contact surfaces by X-ray photoelectron spectroscopy (Quantera SXM system, ULVAC-PHI, Chigasaki, Japan). The chemical composition of the sample surface was revealed in the wide scan spectra. The variation of the atomic composition with film depth was obtained by XPS depth profiling techniques. The film surface was bombarded with Argon ions at controlled power, and the composition was analyzed after every 6 s of the bombard erosion.

## 3. Results and Discussion

### 3.1. Contact Behavior during Contact Making

**Figure 3.**
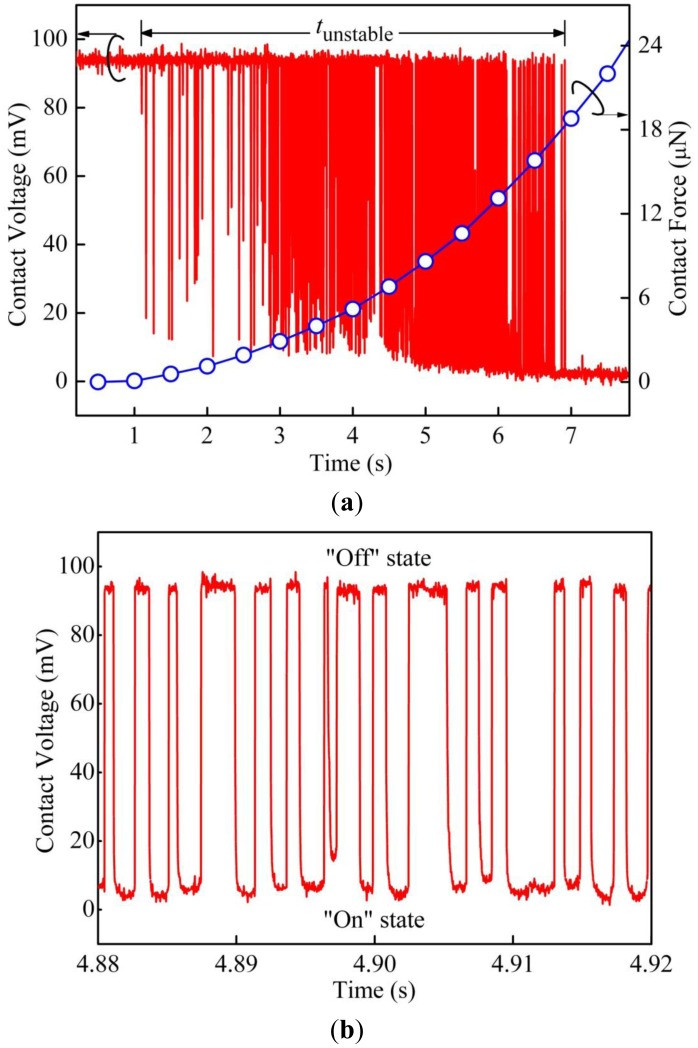
(**a**) Contact voltage and contact force as functions of time for a typical Au-Au contact in Group B; (**b**) Zoom-in from 4.88 to 4.92 s, shows a typical two level random telegraph signal (RTS).

In general, the testing results show similar behavior as reported in references [14,15]. A typical curve of contact voltage *versus* loading time for samples in Group B is shown in Figure 3a.

The contact voltage *V*_c_ was measured between the gold coated ball tip of the piezo-actuator and the sample surface. In this case, the applied voltage was 95 mV. Fast fluctuations of *V*_c_ were captured by the oscilloscope from 1.06 to 6.94 s, before formation of stable electrical contact with *V*_c_ less than 5 mV. Figure 3b zooms in on the time-axis from 4.88 to 4.92 s during the transition period. The switching behavior is similar to a two level random telegraph signal (RTS), which fluctuates between “on” and “off” states. At “on” state, there is current flow between the ball tip and sample surface, and the electrical contact is open at “off” state.

**Figure 4.**
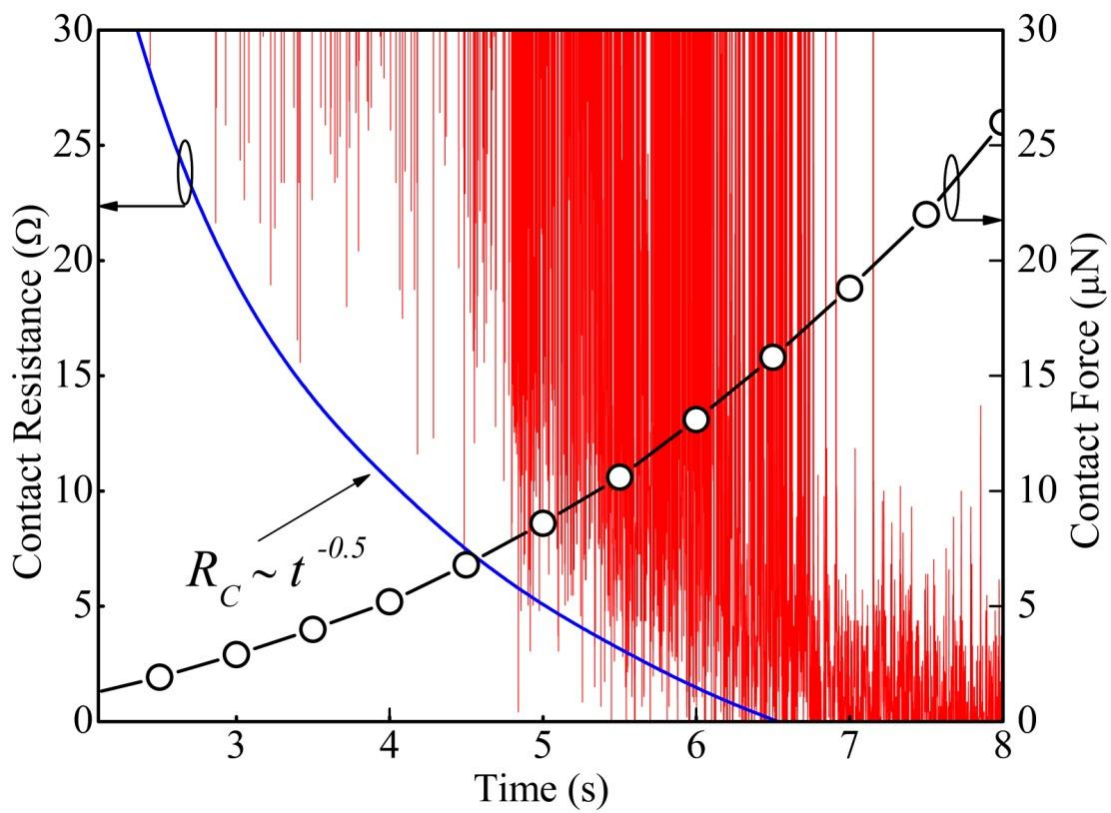
Contact resistance and contact force *versus* loading time. A relation of *R*_c_ ∝ *t*^−0.5^ is illustrated by the dashed line for reference.

Meanwhile, it is interesting to note that, during the fast voltage fluctuations in the unstable region, a decrease in “on” state contact voltage can be observed. It indicates a reduction of electrical contact resistance *R*_c_. The calculated “on” state *R*_c_ in the unstable region is shown in Figure 4, and it drops from around 30 to 1 Ω. In addition, we found that the electrical contact resistance at the “on” state could be well fitted using a relation *R*_c_∼*t*^−^*^n^* with *n* ≈ 0.5. Since the relation between the contact force and tip displacement is almost linear after initial contact and the tip displacement velocity is fixed, therefore, *R*_c_∼*F*_c_^−0.5^ is expected. This strongly suggests that the plastic deformation of the asperities, which was observed in the beginning of the stable contact (Region II) by Kwon *et al.* [14], could start from the very early stage of the contact process even in the unstable region. In this case, the contact force at the beginning of unstable region is ∼250 nN, and the minimum contact force required to reach the stable region is ∼18 μN. The contact force at the transition point from elastic to plastic deformation for a single asperity was calculated to be around 100 nN by Majumder *et al.* [3], and our result is in well agreement with this value.

**Figure 5.**
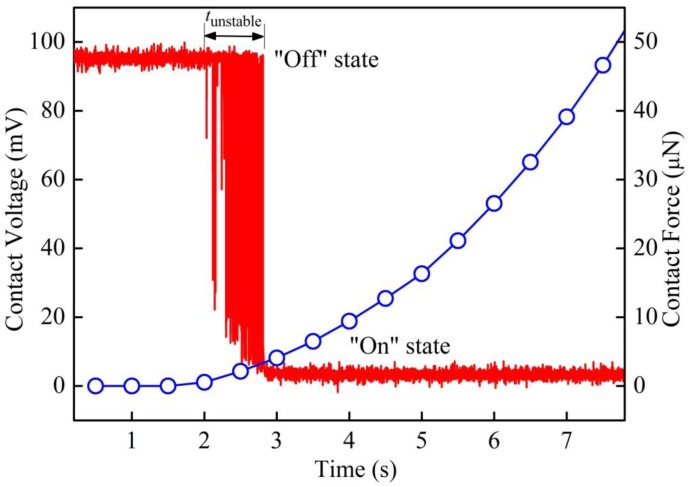
Contact voltage and contact force as functions of time for a typical Au-Au contact in Group A.

For the “fresh” samples in Group A, a typical curve of contact voltage *versus* loading time is shown in Figure 5. Similar to Group B, instability of electrical contact was observed in the low contact force region. In this case, the switching behavior lasts for about 0.8 s, which is remarkably shorter, compared to those samples underwent lithography process. The contact forces at the starting and ending points of the transition region are ∼500 nN and ∼4 μN respectively.

**Figure 6.**
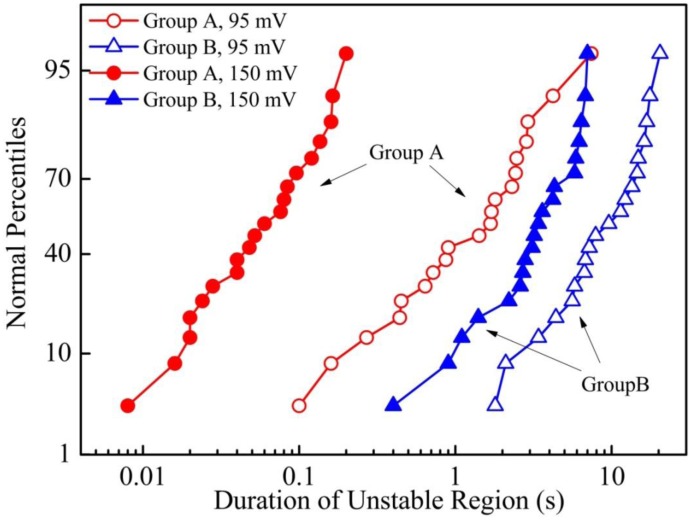
Probability plot of the duration of the unstable region based on 20 independent tests for four combinations of testing conditions (Group A, *V*_C_ = 95 and 150 mV; Group B, *V*_C_ = 95 and 150 mV).

The distribution of the durations of the unstable region for both groups A and B under two applied voltage levels are shown in Figure 6.

Experimental data of 20 independent loading tests are used for each probability plot. It can be seen that samples exposed for one lithography cycle (Group B) have much longer transition periods than those “fresh” samples (Group A). This clearly indicates that the contact behavior at the initial contact stage could be strongly related to the surface contamination during the fabrication process. Moreover, the duration of unstable region is largely affected by the applied contact voltage. A higher *V*_C_ results in a much shorter unstable region. Total annihilation of the fluctuations was observed for *V*_C_ beyond 250 mV during the contact tests. This suggests that fast switching behavior in the unstable region should be associated with the mechanisms related to electrical conduction. The possibility of pure mechanical deformation such as contact bouncing or other external vibrations, which are irrelevant to electrical bias, can be ruled out. In the past, sudden and stochastic changes in the contact resistance profile are often attributed to arcing in switch applications [21].

### 3.2. XPS Analysis of Contact Surfaces

**Figure 7.**
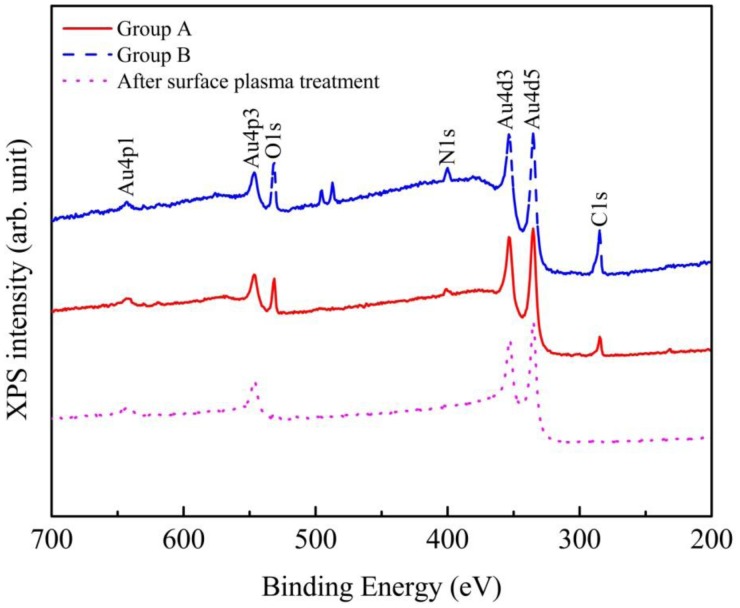
XPS spectra of the samples taken from Group A, Group B and after surface plasma treatment. Alien films containing O, N and C are identified for both samples.

To validate the presence of alien hydrocarbon layer at the contact surfaces, samples from each group were characterized by XPS techniques. It is found that the sample, which underwent one cycle of lithography process, shows a much thicker alien layer with stronger signal intensities for O, N and C. As shown Figure 7, there are three peaks centered at 543.1, 410 and 284.2 eV for the samples, which could be assigned to O 1s, N 1s and C 1s respectively. This layer could be due to the polymer residues induced by the fabrication process or absorption of air-born species [17,21,22]. For the sample from Group B with photoresist residues, stronger O 1s, N 1s and C 1s signals are identified than the case of Group A. The spectrum of the sample after plasma cleaning is also shown in the figure as a reference.

**Figure 8.**
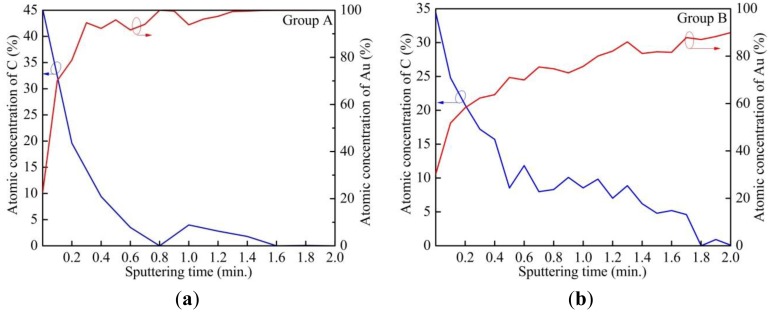
XPS depth profiling (**a**) sample from Group A; (**b**) sample from Group B.

The results of depth profiling using XPS are shown in Figure 8. The percentages of atomic concentrations of C and Au are plotted *versus* sputtering time. The etch rate of polymer (photoresist) is estimated to be 2 nm/min. Using Au percentage of 80% as a reference, the thickness of the alien film is around 0.4 and 2.2 nm for the sample from Group A and Group B, accordingly. The sample exposed after a complete lithography cycle has a thicker alien layer, which is consistent with the stronger O 1s, N 1s and C 1s signals of the XPS spectra in Figure 7.

### 3.3. Mechanism of Contact Resistance Fluctuation

**Figure 9.**
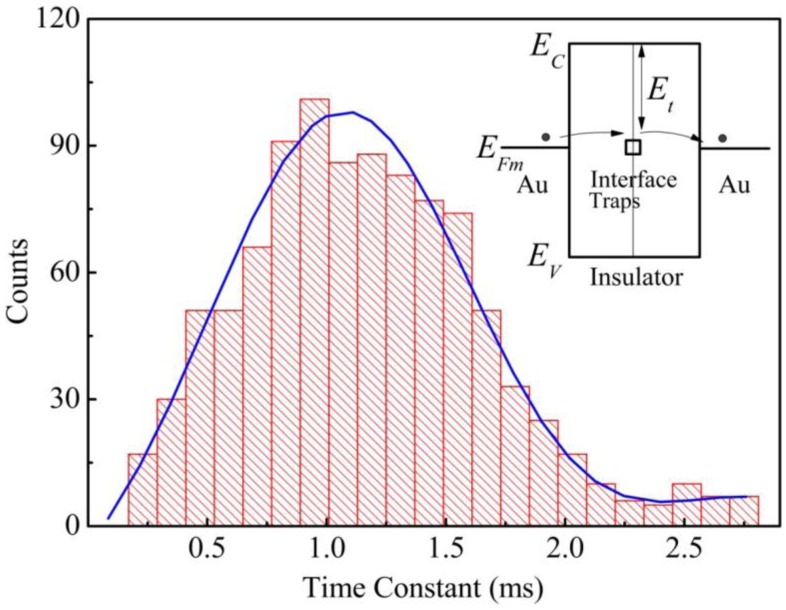
A typical histogram of the time intervals for “off” state for a sample measured at 95 mV. Inset shows the energy band diagram of the Au/Insulator/Au structure. Electrons tunnel though the traps located at the contact interface between the alien films.

Due to the existence of an alien film on the contact surface, the electrical contact may remain non-metallic at the initial contact stage at low contact force. Stable electrical contact only forms when the insulating contamination layer is eventually penetrated mechanically or broken down under current or heat. In the unstable region, electrical conduction can be attributed to tunneling process through the alien films, and traps located at the contact interface may play a very important role in determining the charge transport. The on-off behavior can be explained by the charge trapping and detrapping processes as illustrated in the inset of Figure 9. The “on” state occurs when there are conductions between the Au electrodes due to the electrons tunneling through the insulating layer via the electron traps at the contact interface. Once the traps are occupied, the electrical conductance switches to the “off” state.

To further investigate the fast switching behavior in the unstable region, time intervals of each “off” state were statistically analyzed for Group B. Figure 9 shows a typical histogram of the time intervals extracted from a contact voltage *versus* loading time curve. It can be seen that there is a distinct peak with time constant of approximately 1.1 ms, which could be associated with the trapping and detrapping processes of electrons from the traps located at the contact interface. Considering electron capturing and releasing at the traps with different energy levels located at the contact interface, the time constant (τ) under a small electric field can be estimated by [23]:

(1) $$\tau (x)=\tau_{0}\exp(\frac{4\pi \sqrt{2m_{t}^{*}E_{t}}}{\hbar }x)$$

where τ_0_ is the charge transferring characteristic time, $m_{t}^{*}$ is the electron tunneling effective mass, *E_t_* is the trap energy with respect to the conduction band edge, *ħ* is Planck's constant, and *x* is the trapped charge position. Using τ_0_ = 10^−13^ s [24–26], $m_{t}^{*}=0.4m_{0}$, *ħ*=6.625×10^−34^ J·s, *x* = 2 nm, the corresponding trap energy level at the contact interface is 3.16 eV. If the traps are the mid-band traps located at the center of bandgap, a bandgap of around 6.3 eV for the alien layer will be expected. This is in good agreement with the typical bandgap of the polymers used for microfabrication process such as photoresist and polyimide, *etc.*, [27,28].

It should be noted that pure Au-Au contact in low force region may only happen under certain circumstances for actual MEMS device applications. However, understanding of the role of the alien film on contact surface can be extended to other switch designs with different contact materials, such as Ru, Pt, and AuNi.

## 4. Conclusions

In conclusion, the instability of electrical contact behavior in low contact force region has been investigated and analyzed experimentally for Au-Au micro/nano-contacts. XPS techniques were applied to characterize the contact surface. The results reveal that the alien film plays an important role in determining the unstable behavior at initial contact stage. This is clearly evidenced by the presence of a remarkable transition region on the samples exposed to photoresist, which leads to a much thicker alien hydro-carbon layer. The RTS observed in the unstable region can be explained under a framework of trap-assisted electron tunneling through the alien film.
